# New Oral Anticoagulants in the Treatment of Pulmonary Embolism: Efficacy, Bleeding Risk, and Monitoring

**DOI:** 10.1155/2013/973710

**Published:** 2013-04-10

**Authors:** Kelly M. Rudd, Elizabeth (Lisa) M. Phillips

**Affiliations:** ^1^Department of Pharmaceutical Care Services, Bassett Medical Center, Cooperstown, NY 13326, USA; ^2^Wegmans School of Pharmacy, St. John Fisher College, Rochester, NY 14618, USA

## Abstract

Anticoagulation therapy is mandatory in patients with pulmonary embolism to prevent significant morbidity and mortality. The mainstay of therapy has been vitamin-K antagonist therapy bridged with parenteral anticoagulants. The recent approval of new oral anticoagulants (NOACs: apixaban, dabigatran, and rivaroxaban) has generated significant interest in their role in managing venous thromboembolism, especially pulmonary embolism due to their improved pharmacokinetic and pharmacodynamic profiles, predictable anticoagulant response, and lack of required efficacy monitoring. This paper addresses the available literature, on-going clinical trials, highlights critical points, and discusses potential advantages and disadvantages of the new oral anticoagulants in patients with pulmonary embolism.

## 1. Introduction

Pulmonary embolism (PE) is a common, potentially fatal disease with an annual incidence of approximately 70 cases per 100 000 people [1, 2]. Patients generally require hospitalization and recurrence is common [3]. In addition to significant morbidity and mortality, treatment costs associated with thrombosis and the arising postthrombotic syndromes are staggering, with costs exceeding 500 million dollars annually in the United States [1, 4, 5].

The mainstay of treatment for the past fifty years has been warfarin therapy, overlapped with a parenteral anticoagulant until the vitamin K antagonist (VKA) is fully therapeutic [2, 5]. While highly effective at reducing morbidity and mortality associated with PE, VKA therapy poses challenges, including variability in drug response, patient compliance, and drug-drug, drug-disease, and drug-diet interactions [6–9]. Great interest has been generated regarding the recently marketed new oral anticoagulants (NOACs) and their potential role as alternative anticoagulant therapies.

## 2. Risk Assessment in Pulmonary Embolism

Pulmonary embolism is a serious and potentially fatal complication of a venous thrombotic event (VTE). Despite clinical trials yielding similar estimates for safety and efficacy in overall treatment when comparing deep vein thrombosis (DVT) and PE, patients with PE incur additional risks not seen in patients with DVT alone. The impact of PE on mortality is striking, with significant increases in the risk of death at both 30 and 90 days after event [10]. In addition to increasing mortality, PE also has significant impact on morbidity. Cardiorespiratory impairment, including pulmonary hypertension, can result in chronic and irreversible health concerns. Additionally, recurrent episodes of either VTE or PE are approximately 40% higher in patients with history of a PE, propagating the dangers of VTE and PE into the future [5, 10]. These factors culminate in an essential need for safe and effective anticoagulant treatments for pulmonary embolism.

Three new oral anticoagulants (apixaban, dabigatran, and rivaroxaban) are the newest to enter the anticoagulant armamentarium. The available data on each agent in the treatment of PE, their respective mechanisms of action, pharmacokinetic profiles, and potential places in therapy are discussed. 

## 3. Relevant Pharmacokinetic and Pharmacodynamic Clinical Pearls 

Each of the NOACs has unique pharmacokinetic and pharmacodynamic properties that clinicians must consider when selecting oral anticoagulant therapy. The comparative pharmacokinetic profiles for the NOACs in respect to warfarin are provided in Table 1. The newer agents have a more rapid onset and shorter duration of activity than warfarin. This suggests that standard management of starting a parenteral anticoagulant simultaneous to an oral VKA (bridging) therapy upon initiation or interruption of therapy may become unnecessary in some patients. An important caveat to this change is that some Phase II and III data indicate a potential need for higher doses for some of the NOACs during the first few weeks of treating an acute thrombotic event, requiring close attention to dosing recommendations and adjustments [11, 12]. Clinical trials with dabigatran, however, have utilized a study design requiring a lead-in treatment period with a parenteral anticoagulant prior to the initiation of study medication thereby making a decision about the utility of dabigatran as primary treatment for VTE an unanswered clinical question [13, 14]. The reader is directed to Section 4 of this paper for more details on available clinical trials. 

An equally important point is the dosing schedules of the NOACs range from once to twice daily, despite relatively short half-lives. This makes compliance, appropriate counseling and monitoring imperative components to successful therapy. Although the NOACs do not have the dietary restrictions of warfarin, an important caveat for rivaroxaban is that higher doses (i.e., >10 mg/day) need to be given with food to enhance bioavailability. Additionally, the rivaroxaban package insert recommends avoiding administration via feeding tube due to concerns of decreased bioavailability. While there are no dietary restrictions with dabigatran, the dosage form cannot be crushed or chewed as bioavailability increases well above therapeutic levels. Dabigatran must also be stored in the original container to prevent capsule degradation, with the medication expiring 4 months after opening of the original container. Dabigatran therefore cannot be placed in a pill box or organizer because of its hygroscopic tendencies.

The NOACs have fewer drug interactions than warfarin; however vigilance with patient's medication profiles and concomitant medications is necessary as the NOACs are subject to drug-drug interactions via the cytochrome p450 and p-glycoprotein systems (Table 2). Note that the suggested dosing alterations provided in Table 2 for dabigatran and apixaban are those noted in the package inserts currently approved under the indications of nonvalvular atrial fibrillation (AF). In many cases, the treatment doses utilized for PE are higher than those approved for other indications, making caution necessary when applying available dose adjustment recommendations to patients with VTE. If these agents receive FDA approval for the treatment of PE, updates to the package inserts to address drug interactions and recommendations for adjustment to the newly approved doses will be imperative. As rivaroxaban is now approved for the treatment of PE as well as nonvalvular AFIB and DVT prevention, the table highlights the interactions consistent with these dosing schedules.

Lastly, the varying degree of renal elimination and altered response in patients with evidence of renal dysfunction adds to the clinical complexity for the appropriate patient selection. Dabigatran, rivaroxaban, and apixaban have FDA approved dosing adjustments for patients with nonvalvular AF and who have compromised renal function. For dabigatran and rivaroxaban, these renal dosing recommendations, while FDA-approved, are based upon pharmacokinetic studies and have not been studied clinically. When apixaban was studied, the clinical trials took into account the patients level of renal function and so the dose adjustment in the package insert is based on validated data from the clinical trial. Therefore, the FDA approved dose for apixiban is to reduce the dose in patients with two of the following characteristics: age ≥80 years, body weight <60 kg or serum creatinine ≥1.5 mg/dL (Table 2). Additional considerations are necessary in patients with both renal dysfunction and concurrent drug-drug interactions, as the pharmacokinetics become even more uncertain. Consideration for continued use and referral to a specialized anticoagulation clinic or service offers the prescriber with a comprehensive assessment of the appropriateness of patient selection for the NOACs.

The predictable pharmacokinetic profile of the newer NOACs results in less variability in drug response thereby negating the need for individualized therapeutic monitoring as required with VKA. Although the newer agents can alter coagulation assays (Table 1), there is an inconsistent relationship between drug effect and assay response to allow clinical utility of laboratory monitoring. Although there is a clinically appealing feature of these new agents, this poses a therapeutic challenge when bleeding occurs or urgent reversal is required, as traditional coagulation assays do not provide quantitative evidence for the intensity of anticoagulant effect. However, these assays can be used to qualitatively assess the presence of drug and thereby guide course of action in the event of emergency. Conversely, given the relative insensitivity of traditional assays (such as the INR and aPTT) to the NOACs, normalization of the assay may not always indicate lack of drug or complete reversal of the pharmacologic effect [15, 16].

The lack of a reversal agent for the NOACs remains one of the most important clinical concerns with these new agents. As the NOACs pharmacologically do not induce clotting factor deficiencies as a mechanism of action, the utility of replacement products such as fresh frozen plasma (FFP) is limited. Since the NOACs represent inhibitors of the coagulation cascade, reversal will remain a challenge until an antidote is developed and tested clinically. 

Currently, only summary recommendations regarding the management of bleeding or “reversal” of the NOACs can be made. Although there are published studies, many are ex vivo or animal studies that cannot reliably predict an in-vivo response and therefore recommendations remain inconclusive. In vitro publications in humans are sparse and primarily limited to case reports [17]. For reversal of dabigatran, the use of activated charcoal and dialysis in cases of life threatening bleeding or overdose can be considered [18]. Activated Factor VIIa (Novoseven) is a seemingly attractive option though it has not been studied in humans. In animal models, bleeding complications were not reliably and consistently reversed [19], and use in the nonhemophiliac population has been associated with an increased risk of thrombosis [20].

A nonactivated 4-factor PCC product (Cofact) failed to show reversal in coagulation parameters induced by dabigatran in a randomized, placebo-controlled, crossover study in healthy subjects. In this study, bleeding was not an accessible outcome [15]. van Ryn and colleagues performed an in vitro study using nonactivated 4-factor PCCs (Beriplex and Octaplex), an activated PCC product (FEIBA), and an activated Factor VII (Novoseven) and found a reduced bleeding time after administration, despite failure to reverse coagulation parameters such as aPTT and thrombin time [16]. These animal model studies suggest a potential lack of correlation between changes in coagulation parameters and bleeding for dabigatran, and further study is needed to confirm this hypothesis [15]. 

In regard to rivaroxaban, dialysis is not a viable option due to high protein binding (92–95%) [21], and no data on activated charcoal could be found. Animal studies using activated Factor VII (Novoseven) show only modest effect on reducing bleeding, and there are no human clinical data to support routine use [22]. Additionally, the concern of increased risk of thrombosis remains with activated Factor VII use [19]. A randomized, placebo-controlled, crossover study in healthy subjects suggested that a nonactivated 4-factor PCC product (Cofact) used in healthy adults could reverse rivaroxaban effect on the PT; however it is unknown whether this translates into decreasing bleeding [15]. 

No data or recommendations currently exist for reversal of apixaban therapy. To complicate translation of this research into clinical practice, nonactivated 4-factor PCCs are unavailable in the United States, and the only 4-factor PCC available is the activated FEIBA product. Institutions are therefore left with the development of institution-specific policies for reversal based upon the foundation of supportive care and local measures to stop bleeding, the availability of the local medical expertise, an understanding of available literature and reversal agents, and cost.

## 4. Anticoagulant Therapy for Treatment of Pulmonary Embolism

### 4.1. Apixaban

Limited data exist to determine the safety and efficacy of apixaban for the treatment of PE. The AMPLIFY (NCT00643201) trial is a Phase III, randomized, double-blind, double-dummy, noninferiority trial ongoing to assess apixaban in the initial treatment of VTE [33]. AMPLIFY is currently enrolling patients in a 6-month study, comparing dose-adjusted warfarin (INR goal 2.0–3.0) with parenteral anticoagulant bridge to apixaban 10 mg twice daily for 7 days followed by apixaban 5 mg tablets twice daily. The study is expected to enroll 4816 in 471 study locations and is expected to close in March 2013 with a primary outcome of a composite time to recurrent VTE or death [33]. No safety or efficacy data are presently available from the AMPLIFY trial.

AMPLIFY-EXT is a randomized, double-blind study evaluating the safety and efficacy of apixaban for the extended treatment of DVT and PE over a 12-month period following the initial 6–12 months of anticoagulation therapy [34]. Subjects were randomized to apixaban 2.5 mg or 5 mg twice daily or placebo and were eligible if they were 18 years of age or older, had objective confirmed proximal DVT of the leg(s) or PE, had finished 6–12 months of standard anticoagulant therapy, or had completed treatment with apixaban or enoxaparin and warfarin as part of the AMPLIFY trial, had no symptomatic recurrent of VTE during prior anticoagulant therapy, and had clinical equipoise to continue or cease anticoagulation therapy. Patients were excluded if they had contraindication to continued anticoagulant therapy, had dual antiplatelet therapy, were on aspirin doses greater than 165 mg daily, had elevated liver function tests, or has depressed hematologic markers. 

The majority of patients on apixaban in AMPLIFY-EXT weighed greater than 60 kg (92.6%). Serum creatinines (for dose adjustment, per the package insert) were not provided; though the majority of patients on apixaban (99.8%) had a calculated creatinine clearance greater than 30 mL/min, 91.8% had a calculated creatinine clearance greater than 50 mL/min. Of the subjects on apixaban, 98.3% completed 6–12 months of prior anticoagulation therapy, as opposed to less than 6 months and more than 12 months, though no further information was provided as to the exact discontinuation date of the initial anticoagulant therapy. Patients lost to follow were classified as having the primary outcome event and negative for reaching the primary safety endpoint. Study endpoints are presented in Table 3.

Based upon information provided by the authors of the source article, apixaban 5 mg twice daily lowered the rate of recurrent, nonfatal pulmonary embolism [23], when therapy was extended one year beyond the initial anticoagulation period when compared to placebo. Until the results from AMPLIFY are available, it remains unknown if apixaban is safe and effective in the primary treatment of VTE with pulmonary embolism [33].

### 4.2. Dabigatran Etexilate Mesylate

Dabigatran, an oral direct thrombin inhibitor, has been FDA approved for nonvalvular atrial fibrillation and is also approved in Europe and Canada for VTE prevention. Currently, dabigatran is not approved for the treatment of VTE in Canada, Europe, or the United States of America, and applications to the respective regulatory bodies (EMA, HC, and FDA) have not been submitted. Presently four Phase III clinical trials, RE-COVER, RE-COVER-II, RE-MEDY (NCT00329238), and RE-SONATE (NCT00558259), have been completed to assess the efficacy of dabigatran in the treatment of VTE, with or without PE, though only RE-COVER has published full results [13, 14, 35, 36].

RE-COVER is a randomized, double-blind, double-dummy, noninferiority trial conducted in 228 centers world-wide, enrolling 2564 patients [14]. Patients were eligible if they were 18 years of age or older and had objective confirmed proximal DVT of the leg(s) or PE. Patients were excluded if they had VTE symptoms for more than 14 days, experiencing hemodynamic instability requiring thrombolytic therapy, had recent cardiovascular event, or were at high risk for bleeding.

The design of RECOVER was unique in that all patients received a parenteral anticoagulant (intravenous unfractionated heparin or subcutaneous low molecular weight heparin) for the first 6 days, while warfarin dosing was being adjusted to achieve a target INR of 2-3 by sham INR. After that time, patients continued their blinded randomized assignment of either Dabigatran (150 mg twice daily) or warfarin (dose-adjusted to a target INR of 2.0–3.0). Because parenteral anticoagulation was used during the first week, larger initial dabigatran doses, as seen in other NOACs studies, were not utilized as part of this protocol. 

RE-COVER II was of similar design, enrolling 2568 patients and was conducted to confirm the previous results and gather data sufficient for subgroup analyses [35]. Study endpoints are presented in Table 4.

All RE-COVER study patients on dabigatran had a creatinine clearance above 50 mL/min (mean 105.8 ± 40.7) with a median weight of 84 kg (range: 38–175). The proportion of cancer patients, those with the highest risk of development and morbidity associated with PE, were small in the dabigatran and warfarin groups, 5.0% and 4.5%, respectively [14].

Subgroup analysis did not indicate differences in the primary endpoint in relation to gender, age, or race or those presenting with initial symptomatic PE or active cancer at baseline. Likewise, the rate of recurrent VTE in patients with previous VTE was not statistically different in the two treatment groups (*P* = 0.09), though trended to favor warfarin therapy [14].

Comparison data were not available in RE-COVER II as this was presented in abstract form only. Study authors note that a full publication is pending. 

In RE-COVER, dabigatran 150 mg twice daily was noninferior, but not superior to dose-adjusted warfarin managed to a time in therapeutic range (TTR) of 60 percent. The rate of recurrent PE was higher in the dabigatran group (1% versus 0.6%, HR 1.85, 95% CI 0.74–4.64), though not statistically different. The dabigatran 150 mg twice daily dose was also utilized in the RE-LY atrial fibrillation study. Noting that the 18 113 patients were not randomized to correct for VTE risk factors, nor were they reported, the rates of PE development in the dabigatran and warfarin groups were 0.15% per year and 0.09% per year respectively (Relative Risk 1.61, 95% CI 0.76–3.42, *P* = 0.21, warfarin mean TTR 64%) [14].

The RE-MEDY (NCT00329238) and RE-SONATE (NCT00558259) trials have both been completed, with data collection terminating in October 2010 and February 2011, respectively. RE-MEDY is a randomized, double-blind, multicenter, parallel-group, active controlled study to compare the efficacy and safety of dabigatran 150 mg twice daily to dose-adjusted warfarin (INR target 2.0–3.0) for the secondary prevention of VTE. A total of 2867 patients in 275 study locations were followed for a total of 36 months to the primary outcome of composite recurrent VTE or VTE death at 18 and 36 months [13].

RE-SONATE is a randomized, double-blind, multicenter, parallel-group, prevention study to compare dabigatran 150 mg twice daily versus matching placebo in the long-term prevention of recurrent, symptomatic VTE in patients with history of DVT or PE who completed 6–18 months of treatment with VKA therapy. Total study enrollment is 1353 patients in 147 study locations. The primary study endpoint is time to centrally confirmed VTE over the 6-month study period [36].

Bleeding remains a main safety concern with dabigatran and the NOACs. In the setting of acute VTE, warfarin showed slightly higher rates of nonmajor bleeding, though no difference in major bleeding outcomes was found [14]. Despite the lack of difference in clinical trials, Phase IV data with the dabigitran 150 mg twice daily utilized for stroke prevention in AF still raise bleeding as a significant concern. On December 7, 2011, the FDA initiated an investigation into serious bleeding events associated with dabigatran [37]. Likewise, the Institute for Safe Medication Practices reported that the FDA received nearly three times as many serious and fatal side effects in 2011 with dabigatran as compared to warfarin (3781 versus 1106.) This report also concluded that dabigatran surpassed all other regularly monitored drugs in categories such as deaths (542), hemorrhage (2,367), acute renal failure (291), and stroke (64) and was suspected in 15 cases of liver failure [38].

### 4.3. Rivaroxaban

Rivaroxaban, an oral direct Factor Xa inhibitor, has been FDA approved for both nonvalvular atrial fibrillation and VTE prevention and in Canada, Europe, and the United States for VTE treatment. Presently, three Phase III clinical trials, EINSTEIN-DVT, EINSTEIN-PE, and EINSTEIN-Extension, have been completed to assess the efficacy of rivaroxaban treatment in VTE [11, 39, 40].

EINSTEIN-DVT was an open label, randomized, multicenter, noninferiority trial enrolling 3449 patients with symptomatic DVT without PE [11]. Patients were eligible if they were 18 years of age or older, had objectively confirmed proximal deep vein thrombosis (DVT), and were without symptomatic pulmonary embolism. Patients were excluded if they received therapeutic doses of low-molecular weight heparin, fondaparinux, or unfractionated-heparin for more than 48 hours or if they had received more than a single dose of a vitamin K antagonist before randomization, if they had been treated with thrombectomy, a vena cava filter, or a fibrinolytic agents for the current episode of thrombosis, or if they had a contraindications listed in the labeling of the medications used, another indication for warfarin, creatinine clearance <30 mL/min; clinically significant liver disease or an alanine aminotransferase level that was three times the upper limit of normal, active, or high risk for bleeding, bacterial endocarditis, systolic blood pressure greater than 180 mm Hg, or diastolic blood pressure greater than 110 mm Hg; child bearing potential without proper contraception, pregnancy, or breast-feeding; concomitant use of strong cytochrome P450 3A4 inhibitors; participation in another study within 3 days before screening; and life expectancy of less than 3 months. 

In EINSTEIN-DVT, patients were either randomized to either rivaroxaban (15 mg orally twice daily for 3 weeks, followed by 20 mg daily for 3 to 12 months) or a parenteral anticoagulant (enoxaparin 1 mg/kg of body weight subcutaneously twice daily) overlapping with a vitamin K antagonist such as warfarin or acenocoumarol (dose-adjusted to a target INR of 2.0–3.0) [11]. Parenteral anticoagulation was continued for at least five days until an INR above 2.0 was achieved for two consecutive days. Study endpoints are presented in Table 4. In EINSTEIN DVT, rivaroxaban was noninferior (*P* < 0.001) but not superior (*P* = 0.08) to dose-adjusted warfarin/acenocoumarol managed to a time in TTR of 57.7% for the primary endpoint of symptomatic recurrent VTE, defined as the composite of DVT or nonfatal or fatal PE. Efficacy was maintained throughout the study, even during the time of transition from twice daily dosing to once daily dosing for rivaroxaban and across all prespecified subgroups. Additionally, the net clinical benefit, taking into account the primary efficacy and safety outcomes, statistically favored rivaroxaban over parenteral anticoagulants plus VKA (2.9% versus 4.2%, resp.; *P* = 0.03). These endpoints were achieved with no additional risk of major bleeding or the primary composite safety endpoint of major bleeding or clinically relevant nonmajor bleeding (Table 5).

EINSTEIN-PE was an open label, single-blinded (outcomes assessment only), randomized, multicenter, noninferiority trial enrolling 4833 patients with symptomatic PE with or without DVT [39]. Inclusion and exclusion criteria were similar to EINSTEIN DVT as were the treatment regimens. The intended duration of treatment was determined by the treating physician before randomization but was either 3 months, 6 months, or 12 months. Study endpoints are presented in Table 4. In EINSTEIN-PE, after a mean study treatment duration of approximately 7.2 months in both groups, rivaroxaban was noninferior (*P* < 0.003), but not superior (*P* = 0.57) to dose-adjusted warfarin/acenocoumarol managed to a time in TTR of 62.7% for the primary endpoint of symptomatic recurrent VTE, defined as the composite of DVT or nonfatal or fatal PE. Efficacy was maintained throughout the study, even during the time of transition from twice daily dosing to once daily dosing for rivaroxaban and across all prespecified subgroups such as age, sex, presence of absence of obesity, level of renal function, or extent of pulmonary embolism. However, unlike EINSTEIN-DVT, there was no statistical difference in the net clinical benefit taking into account the primary efficacy and safety outcomes between the two groups (Table 4) [11]. These endpoints were achieved with no statistically significant increase in the risk of the primary safety outcome defined as the combination of the first major or clinically relevant nonmajor bleeding episodes but with a statistically significant decrease in the risk of major bleeding between rivaroxaban versus parenteral anticoagulant plus VKA (1.1% versus 2.2%  HR = 0.85 (CI: 0.31–0.79); *P* = 0.003).

EINSTEIN-Extension study was a placebo-controlled, double-blinded, superiority trial designed to determine if extended therapy would decrease the risk of recurrent VTE in patients with confirmed DVT or PE previously treated for 6–12 months with either VKA or rivaroxaban [40]. A total of 1196 patients were enrolled into the trial with 34.1% of the patients coming from the EINSTEIN DVT study and 19.1% completing the EINSTEIN PE study while the remaining 47.5% were referred from outside these studies. Patients were randomized to receive either rivaroxaban 20 mg orally daily or placebo for a duration of 6–12 months. Baseline characteristics were similar between groups. Not surprisingly, rivaroxaban was shown to be superior to placebo in the primary outcome of the prevention of recurrent VTE with a relative risk reduction of 82% (1.3% versus 7.1%, resp., HR = 0.18 (95% CI 0.09–0.39) *P* < 0.001). The endpoint was achieved without increasing the risk of the primary safety outcome defined as major bleeding (0.7% versus 0%; HR = not reported; *P* = 0.11). However the incidence of the composite endpoint of major bleed or clinically relevant nonmajor bleeding occurred statistically more often in the rivaroxaban group versus placebo (6% versus 1.2%; HR = 5.19 (95% CI 2.3–11.7); *P* < 0.001). Additionally, the net clinical benefit, taking into account the primary efficacy and safety outcomes, statistically favored rivaroxaban over placebo (2% versus 7.1%; HR = 0.28 (95% CI 0.15–0.53; *P* < 0.001). Overall, extended therapy prevented 34 recurrent events at the cost of 4 major bleeding events, nonfatal and an increased risk of clinically relevant nonmajor bleeding events compared to placebo.

The EINSTEIN studies, although large and well executed, have the limitation of an open label design lending toward potential bias, although the investigators have indicated that this was unlikely to bias in favor of rivaroxaban. Additionally the use of a placebo control for the extension trial provides little assistance in comparing it against existing therapy, warfarin, and its relatively short duration of follow-up (mean of 9 months) does not clearly address safety and efficacy for years of chronic therapy. Although secondary analyses of the EINSTEIN studies did not identify any signals or concern for liver toxicity or evidence of unexpected thrombotic events, longer duration of therapy to address a chronic lifelong condition would have been valuable [11, 40]. The most recent update of the ACCP Guidelines on Antithrombotic Therapy from 2012 [2] suggests extending warfarin therapy beyond 3 months in patients with unprovoked VTE who have low-to-moderate risk of bleeding, and if bleeding risk is high, then therapy is limited to 3 months. Having a trial that compares the bleeding risk of a new agent against standard VKA therapy during extension of therapy would help refine these recommendations even further, especially if bleeding risk is lower with the NOACs compared to VKA. The EINSTEIN studies included a high percentage of unprovoked events (range between 60% and 70%) and 4–6% of patients with active cancer making this a moderate-to-high risk population for recurrent events that could have effectively addressed many of these questions. Further research is therefore recommended for the chronic use of rivaroxaban for VTE prevention after an initial event. 

## 5. Conclusions

Based upon the data presented, dabigatran and apixaban cannot be recommended until further safety and efficacy data are available. Rivaroxaban however, represents the most promising of the NOACs for treatment of PE and is indicted in Canada and Europe for this indication and recently received FDA approval for this indication [21]. Although the ACCP 2012 Guidelines on Antithrombotic Therapy published in February 2012 indicated warfarin as the agent with the most sufficient data, the results of the EINSTEIN PE were not yet available at the time of publication [2]. With the approval of rivaroxaban for treatment of VTE (DVT and PE) now in the USA, Canada, and Europe, this offers yet another treatment option. However, selection of rivaroxaban over warfarin must take into account a full assessment of an individual's renal function, concomitant drug therapy, and risk for bleeding. Although rivaroxaban was shown to be noninferior to warfarin in both the DVT and PE studies [11, 40], the risk of major bleeding was statistically lower only in the PE study [40]. The issue of bleeding risk is paramount in the decision of appropriate patient selection due to the lack of reversal agent. Comparative efficacy is also warranted in the setting of specialized anticoagulation clinics where the TTR is generally above 70 percent [41–45] as the current studies achieved only a 57–62% TTR. Lastly, long-term (>12 months) comparison data against standard warfarin therapy are lacking, and therefore recommendations for extended therapy beyond the acute phase of treatment cannot be made. 

Based on the strength of available evidence, warfarin proves to be a monitorable, reversible, and effective agent in patients across the spectrum of concurrent disease and should remain the first-line option in conjunction with appropriate parenteral anticoagulation for the treatment of pulmonary embolism, especially in patients who require chronic therapy or therapy beyond 3 months. However, rivaroxaban represents a viable alternative in patients with less than optimal INR control (TTR < 60%) or in whom warfarin monitoring and management is not possible. 

## Figures and Tables

**Table 1:** Comparison of the pharmacokinetic and pharmacodynamic features of oral anticoagulant therapies [15, 18, 21, 23–31].

Characteristic	Warfarin	Apixaban	Dabigatran	Rivaroxaban
Mechanism of action	VKORC1 enzyme inhibitor	Direct^*∧*^ Factor Xa inhibitor	Direct^*∧*^ Factor IIa (Thrombin) inhibitor	Direct^*∧*^ Factor Xa inhibitor

Prodrug	No	No	Yes	No

Approved indications	VTE prevention VTE treatment Atrial fibrillation Valvular heart disease (all approved in EMA, HC, FDA)	VTE prevention (EMA, HC) Nonvalvular AF (EMA, HC, FDA)	VTE prevention (EMA, HC) Nonvalvular AF (EMA, HC, FDA)	VTE prevention (EMA, HC, FDA) Nonvalvular AF (EMA, HC, FDA) VTE treatment (EMA, HC, FDA)

FDA black box warnings	Agent can cause major or fatal bleeding; Regular INR monitoring is necessary; drugs, dietary changes, and other factors affect INR levels (FDA)	Discontinuation in patients without adequate continuous anticoagulation increases the risk of stroke (FDA)	None to date	Discontinuing places patients at an increased risk of thrombotic events; Risk of spinal/epidural hematoma during neuraxial anesthesia/spinal puncture (FDA)

Dosing	Variable, patient specific	Fixed, twice daily With dose reduction in two of the following patients: (per FDA) (1) Age ≥ 80 years (2) Weight ≤ 60 kg (3) Serum creatinine ≥1.5 mg/dL	Fixed, twice daily With dose reduction in patients with renal dysfunction (per FDA)	*VTE prevention and nonvalvular AF*: Fixed, once daily With dose reduction in patients with renal dysfunction (per FDA) *VTE treatment*: Fixed, twice daily × 3 weeks then fixed, once daily Not to be used if ClCr <30 mL/min

*T*_max⁡_ (h)	4	1–3	1–3	2–4

Half-life (h)	20–60	12	12–17	5–9

Bioavailability (F)	100%	50%	6% (~75% if capsule opened)	10 mg: 80–100% (regardless of food) 20 mg: 66% (without food). Food increases absorption^#^

Renal Clearance	>90% as inactive metabolites	25%	80%*	36% unchanged drug* 33% as inactive metabolites Mostly via secretion not glomerular filtration

CYP metabolism^+^	>90% (S-enantiomer is a substrate for 2C9 and 2C19 while R-enantiomer is a substrate for 1A1, 1A2, and 3A4 )	15% (primary substrate for CYP3A4; minor contributions by others, without active metabolites)	No	30% (substrate for CYP3A4, CYP2J2)

P-glycoprotein (P-gp)^+^	No	Yes	Yes	Yes

Dietary considerations	Dietary vitamin K	None	None	*VTE prevention dose*: does not specify *AFIB*: Take with evening meal VTE treatment: Take with food

Influence on routine coagulation assay				
Protime (PT)	↑	↑	↑	↑

aPTT	No/↑	↑	↑	↑

Thrombin time (TT)	No	No	↑↑	No

Coagulation assay used to monitor efficacy	INR (Protime)	None	None	None

Clinically validated Reversal agent	Vitamin K, FFP, PCC, Factor VIIa, aPCC	None	None	None

EMA: European Medicines Agency; HC: Health Canada; FDA: U.S. Food and Drug Administration

AF: Atrial Fibrillation; CYP: cytochrome P450; VKORC1: C1 subunit of the vitamin K epoxide reductase enzyme; FFP: Fresh Frozen Plasma; PCC: Prothrombin Complex Concentrate.

^*∧*^Does not bind to antithrombin.

^#^Rivaroxaban 15 mg tablet, if available, should be taken with food.

*Dose adjustment for level of renal dysfunction required.

^+^Potential source for drug-drug interactions—review full package insert for details.

**Table 2:** Selected P-glycoprotein and Cytochrome P450 3A4 drug interactions with NOAC based on current FDA-approved indications [18, 21, 31, 32].

Medications^+^	Mechanism of Interaction	Dabigatran^#^ (Pradaxa)	Rivaroxaban^*∧*^ (Xarelto)	Apixaban (Eliquis)
Rifampin	P-glycoprotein inducer	Avoid combination	Avoid	Avoid

Carbamazepine, phenytoin, St. John's wort	P-glycoprotein inducers and Strong 3A4 inducers	Avoid	Avoid	Avoid

Dronedarone	P-glycoprotein inhibitors	*For CrCl 30–50 mL/min:* Dose reduction suggested in dosing for Atrial Fibrillation *For CrCl < 30 mL/min*: Aviod	Not addressed in Package Insert	Not addressed

Systemic ketoconazole Itraconazole	P-glycoprotein inhibitors and Strong 3A4 inhibitors	*For CrCl 30–50 mL/min*: Dose reduction suggested in dosing for Atrial Fibrillation	Avoid	Reduce dose in 1/2 for those starting on the full dose of 5 mg BID^%^

lopinavir/ritonavir, ritonavir, indinavir/ritonavir, conivaptan	P-glycoprotein inhibitors and Strong 3A4 inhibitor	Not addressed in Package Insert	Avoid	Reduce dose in 1/2 for those starting on the full dose of 5 mg BID^%^

Verapamil, amiodarone, quinidine,	P-glycoprotein inhibitors	*For CrCl 30–50 mL/min*: No dose adjustment needed, monitor clinical course *For CrCl < 30 mL/min*: Aviod	Not addressed in Package Insert	Reduce dose in 1/2 for those starting on the full dose of 5 mg BID^%^

Clarithromycin	P-glycoprotein inducers and strong 3A4 inducers	*For CrCl < 30 mL/min* Avoid *For CrCl 30–50 mL/min*: No dose adjustment needed, monitor clinical course	Unclear: refer to package insert.	Reduce dose in 1/2 for those starting on the full dose of 5 mg BID^%^

CrCl: creatinine clearance as determined by Crockcoft-Gault equation.

^#^Dabigatran etexilate (prodrug) uses the p-glycoprotein transport system. It is not a substrate, inducer, or inhibitor of the cytochrome p450 system.

^*∧*^Rivaroxaban is a substrate for cytochrome p450 as well as p-glycoprotein.

^+^Listed medications are representatives of potent 3A4 inhibitors and may not be comprehensive.

^%^Those patients who need to start at 2.5 mg BID should avoid this combination.

**Table 3:** Outcomes of apixaban in AMPLIFY-EXT [34].

Outcome	Apixaban 2.5 mg (*n* = 840; 13 LTF)	Apixaban 5 mg (*n* = 813; 20 LTF)	Placebo (*n* = 829; 19 LTF)
Primary efficacy endpoint: composite of symptomatic recurrent vte or death from any cause	32 (3.8%)	34 (4.2%)	96 (11.6%)
	RR versus placebo	RR versus placebo	
	0.33 (0.22–0.48)	0.36 (0.25–0.53)	

Secondary efficacy endpoint: symptomatic recurrent VTE or death related to VTE	14 (1.7%)	14 (1.7%)	73 (8.8%)
	RR versus placebo	RR versus placebo	
	0.19 (0.11–0.33)	0.20 (0.11–0.34)	

Additional endpoint added after trial initiation: composite of symptomatic recurrent VTE, death related to VTE, myocardial infarction, stroke, or death related to cardiovascular cause	18 (2.1%)	19 (2.3%)	83 (10%)
	RR versus placebo	RR versus placebo	
	0.21 (0.13–0.35)	0.23 (0.14–0.38)	

Primary safety endpoint: major bleeding^*∧*^	2 (0.2%)	1 (0.1%)	4 (0.5%)
	RR versus placebo	RR versus placebo	
	0.49 (0.09–2.64)	0.25 (0.03–2.24)	

Secondary safety endpoint: major or clinically relevant nonmajor bleeding^*∧∧*^	27 (3.2%)	35 (4.3%)	22 (2.7%)
	RR versus placebo	RR versus placebo	
	1.2 (0.69–2.10)	1.62 (0.96–2.73)	

Recurrent fatal PE or death where PE could not be excluded	2 (0.2%)	3 (0.4%)	7 (0.8%)
	RR versus placebo	RR versus placebo	
	0.28 (0.06–1.35)	0.43 (0.11–1.68)	

Recurrent nonFatal PE	8 (1%)	4 (0.5%)	15 (1.8%)
	RR versus placebo	RR versus placebo	
	0.53 (0.22–1.23)	0.27 (0.09–0.82)	

Study conclusion	Extension with 12 months of apixaban 2.5 mg or 5 mg twice daily therapy reduced the risk of recurrent VTE and recurrent nonfatal PE, without increasing the risk of major bleeding.		

NR: not reported, LTF: lost to follow (classified as having the primary efficacy endpoint).

^*∧*^Major bleeding as defined in AMPLIFY-EXT: clinical overt bleeding, with associated fall in hemoglobin of at least 2 g per deciliter, the need for transfusion of 2 or more units of red blood cells, involving a critical site or was fatal.

^*∧∧*^Nonmajor bleeding as defined in AMPLIFY-EXT: bleeding that did not meet criteria for major bleeding but AHT was associated with the need for medical intervention, unscheduled contact with a physician, interruption or discontinuation of the study drug, or discomfort or impairment of activities of daily living. In addition, any bleeding compromising hemodynamics, leading to hospitalization, development of subcutaneous hematoma larger than 25 cm^2^ or 100 cm^2^ if traumatic cause, intramuscular hematoma documented by ultrasonography, epistaxis of more than 5 minutes duration, or was repetitive, spontaneous gingival bleeding, spontaneous macroscopic hematuria lasting great than 24 hours, macroscopic gastrointestinal hemorrhage, and hemoptysis not occurring with PE.

**Table 4:** Outcomes of dabigatran in VTE studies [14, 35].

Outcome	RE-COVER (dabigatran versus warfarin)	RE-COVER II (dabigatran versus warfarin)
Primary efficacy endpoint: recurrent symptomatic VTE or VTE-Related Death	2.4% versus 2.1%, HR 1.10 (95% CI 0.65–1.84)	2.4% versus 2.2%, HR 1.08 (95% CI 0.64–1.8)
Secondary efficacy endpoint: recurrent, nonfatal PE	1.0% versus 0.6%, HR 1.85 (07.4–4.64)	NR
Total days overlap between warfarin/dabigatran and parenteral anticoagulant	5–10 days (mean = 10 days)	5–11 days (mean NR)
Major Bleeding^*∧*^	1.6% versus 1.9%, HR 0.82 (95% CI 0.45–1.48)	1.1% versus 1.7%, HR 0.69 (95% CI 0.36–1.32)
Major or clinically relevant non-major bleeding^*∧∧*^	5.6% versus 8.8%, HR 0.63 (95% CI 0.47–0.84)	NR
Any bleeding	16.1% versus 21.9%, HR 0.71 (95% CI 0.59–0.85)	15.6% versus 22.1%, HR 0.67 (95% CI 0.56–0.81)
Death	1.6% versus 1.7%, HR 0.95 (0.53–1.79)	1.9% in both groups
Warfarin TTR	60%	NR
Study conclusion	Dabigatran is noninferior to warfarin Dabigatran is not superior to warfarin	Dabigatran is noninferior to warfarin Dabigatran is not superior to warfarin

NR: not reported, TTR: time in therapeutic range.

^*∧*^Major bleeding as defined in RE-COVER: Clinical overt bleeding, with associated fall in hemoglobin of at least 20 g per liter, the need for transfusion of 2 or more units of red blood cells, involving a critical site or was fatal.

^*∧∧*^Minor bleeding as defined in RE-COVER: spontaneous skin hematoma of at least 25 cm^2^, spontaneous nose bleed of more than 5 minutes in duration, macroscopic hematuria lasting more than 24 hours, spontaneous rectal bleeding, gingival bleeding of greater than 5 minutes, bleeding leading to hospitalization and/or requiring surgical treatment, bleeding leading to transfusion of less than 2 units of red blood cells, or any other clinically relevant bleeding per the investigator.

**Table 5:** Outcomes of rivaroxaban in VTE Studies [11, 40].

Outcome	EINSTEIN-DVT (rivaroxaban versus VKA + parenteral )	EINSTEIN-PE (rivaroxaban versus VKA + parenteral agent)
Primary efficacy endpoint: noninferiority		
Recurrent symptomatic VTE	2.1% versus 3.0%,	2.1% versus 1.8%,
	HR = 0.68 (95% CI 0.44–1.04)	HR = 1.12 (95% CI 0.75–1.68)
	P < 0.001	P = 0.003
# recurrent events that were any type of PE	25 versus 24	32 versus 27

Secondary efficacy endpoint:		
All-cause mortality	2.2% versus 2.9%	2.4% versus 2.1%
	HR = 0.67 (95% CI 0.44–1.02)	HR = 1.13 (95% CI 0.77–1.65)
	*P* = 0.06	*P* = 0.53
Net clinical benefit	2.9% versus 4.2%	3.4% versus 4.0%
	HR = 0.67 (95% CI 0.47–0.95)	HR = 0.85 (95% CI 0.63–1.14)
	*P* = 0.03	*P* = 0.28

Primary safety outcome:		
Major bleeding or clinically relevant nonmajor bleeding	8.1% versus 8.1%	10.3% versus 11.4%
	HR = 0.97 (99% CI 0.76–1.22)	HR = 0.90 (95% CI 0.76–1.07)
	*P* = 0.77	*P* = 0.23
Major bleeding^*∧*^	0.8% versus 1.2%	1.1% versus 2.2%
	HR = 0.65 (95% CI 0.33–1.30)	HR = 0.49 (95% CI 0.31–0.79)
	*P* = 0.21	*P* = 0.003
Clinically relevant nonmajor bleeding^*∧∧*^	7.36% versus 7.0%	9.5% versus 9.8%
	(statistics NR)	(statistics NR)
Warfarin TTR	57.7%	62.7%
Study conclusion	Rivaroxaban is noninferior to warfarin. Rivaroxaban is not superior to warfarin.	Rivaroxaban is noninferior to warfarin. Rivaroxaban is not superior to warfarin

NR: not reported, TTR: time in therapeutic range.

^*∧*^Major bleeding as defined in EINSTEIN: Clinical overt bleeding, with associated fall in hemoglobin of at least 2.0 g per deciliter, the need for transfusion of 2 or more units of red blood cells, involving a critical site or was fatal.

^*∧∧*^Clinically relevant nonmajor bleeding as defined in EINSTEIN: overt bleeding that did not meet the criteria for major bleeding but was associated with medical intervention, unscheduled contact with a physician, interruption or discontinuation of a study drug or discomfort, or impairment of activities of daily life.
